# Hope for Romantic Relationships: Validation of a Brief Scale

**DOI:** 10.3390/bs16050775

**Published:** 2026-05-14

**Authors:** Marius Marici, Lucian Marina, Viorica-Cristina Cormoș

**Affiliations:** 1 Faculty of Psychology and Educational Sciences, Stefan cel Mare University, 720229 Suceava, Romania; 2 Department of Social Sciences, “1 Decembrie 1918” University of Alba Iulia, 510009 Alba Iulia, Romania; 3 Department of Humanities and Social-Political Sciences, Stefan cel Mare University, 720229 Suceava, Romania; cristina.cormos@atlas.usv.ro

**Keywords:** hope for romantic relationships, scale validation, psychometric properties, reliability, construct validity, relationship commitment

## Abstract

This study aimed to develop and validate a brief measure of hope for romantic relationships (HRR), conceptualized as a future-oriented evaluation of relational continuity and quality. The sample consisted of 525 participants. Exploratory and confirmatory factor analyses supported a unidimensional structure, with strong factor loadings and good model fit. The scale showed good internal consistency and construct validity, being strongly associated with commitment while remaining distinct. Incremental validity analyses indicated that hope explained additional variance in psychological aggression and control of personal life beyond commitment, emerging as the strongest predictor. No significant effect was found for pre-supplementing behaviors. These findings support hope as a distinct construct reflecting expectations about the relationship’s future and suggest that the scale is a brief and reliable tool for assessing relational dynamics.

## 1. Introduction

Romantic relationships are shaped by a complex interplay of cognitive, emotional, and motivational processes that influence their development and stability over time (Marici, 2025b; Marici et al., 2023). Among these, future-oriented cognitions play a central role in guiding individuals’ expectations, emotional investment, and behavioral engagement within the relationship (Murray et al., 1996; Simpson, 2007).

## 2. Hope for Romantic Relationships

Within this framework, hope represents a central dimension which is associated with positive outcomes in romantic relationships (Ripley & Worthington, 2014). Hope has been most often conceptualized as a future-oriented cognitive–motivational construct reflecting individuals’ projections regarding goal attainment and their perceived capacity to achieve desired outcomes. Put simply, drawing on C. R. Snyder’s hope theory, hope includes agency (goal-directed energy) and pathways (planning routes to goals) (Snyder, 2002; Rusbult et al., 1998). In the context of romantic relationships, hope may refer to individuals’ beliefs about the continuity, stability, and long-term decision to stay in the relationship. Furthermore, contemporary models emphasize additional facets, including relational agency, relational pathways, and relational aspirations, reflecting motivational processes, perceived strategies, and emotionally valenced anticipation of future relationship outcomes (Shimshock & Le, 2022).

However, hope has often been implicitly embedded within broader concepts such as optimism or commitment (Sternberg, 1986). The triangular love scale focuses mainly on the volitional aspect of commitment or love as a decision. The items refer to permanence, long-term orientation, resistance to difficulties, loyalty and responsibility, and even conscious decision-making. An item closely related to hope is “*I expect my love for _____ to last for the rest of my life.*”. Another commitment measure (Rusbult et al., 1998, 2009) asks romantic participants to describe their feelings regarding five statements (items) referring to the future of the relationship. Items refer to talking about the future, imagining life together in the future, making plans by considering the effect on the relationship, thinking about the future of the relationship or looking for a long-term relationship with the romantic partner. It is obvious that these concepts enclose the idea of hope too. The relationship quality (RQ) scale (Chonody et al., 2018) has an item directly asking lovers to indicate their level of commitment, while another item tests how much one thinks of their partner as a soulmate. The LOT-R scale, although concerning trait optimism, includes items referring to general expectations in relationships: “*Overall, I expect more good things to happen to me than bad*”.

While commitment and hope are related, they represent distinct psychological processes. Commitment reflects a decision to remain in the relationship, which may be driven not only by positive expectations, but also by constraints such as a lack of alternatives, investments, or social pressures. In contrast, hope captures a future-oriented cognitive–emotional evaluation of the relationship, reflecting the extent to which individuals believe the relationship will develop positively (Kiesler, 1977). Importantly, commitment can be maintained even in the absence of hope, whereas hope reflects an active expectation of relational growth and continuity. This distinction highlights that hope represents a qualitatively different dimension of relationship functioning, beyond the decision-based nature of commitment. Love and Rejection Messages Theory (LRM^T^) (Marici, 2025b) claims that the flux of love messages is based on the belief that the relationship needs permanent and constant signs of care to function properly. LRM^T^ aligns with growth beliefs by conceptualizing relationships as dynamic and improvable through natural processes, with conflicts viewed as opportunities for development. In contrast, destiny beliefs (Knee, 1998) emphasize fixed compatibility and are associated with lower resilience when facing relational challenges. Thus, the aspirational aspect of the hope construct may be viewed as the best theoretical fit for a brief and time-efficient measure in interventional contexts (therapeutic or social assistance), owing to its strong future-orientation. Expectations, which can take the form of cognitive or emotional representations of the partner or of the romantic relationship, play a central role in shaping relationship dynamics. Research suggests that individuals who maintain positively biased perceptions of their partners are more likely to experience higher levels of long-term relational persistence (Murray et al., 1996; Rusbult, 1983). These cognitive appraisals function as interpretative filters that guide emotional responses and behavioral investments within the relationship (Reis, 1990; Finkel et al., 2017), thereby reinforcing relational continuity over time. The way partners are cognitively represented is not merely descriptive, but actively contributes to the construction of relationship quality (Tidwell et al., 2013). In this context, hope has even been theorized as the center of a therapeutic intervention (Ripley & Worthington, 2014).

Although less studied, hope is considered a fundamental element in romantic relationship functioning. Hope theory (Snyder, 2002) claims that hope is a cognitive–motivational system, which implies goals, routes or agency that orient actions to desired relationship outcomes. Positive Illusion Theory (Murray et al., 1996) suggests that positively biased cognitions act as psychological buffers that sustain commitment over time. Love and Rejection Messages Theory (Marici, 2025b) conceptualizes relationship functioning as a dynamic system of exchanged affective messages between partners, grounded in the premise that individuals primarily engage with each other as a result of the expectations of the role of lovers. Relationship outcomes are shaped by mutual dependence and cognitive evaluations, or rewards and costs (Van Lange & Balliet, 2015), which create expectations. The Investment Model of Commitment (Rusbult et al., 1998) explains commitment as a function of satisfaction, the quality of alternatives and the investment in the relationship. Therefore, hope may represent a complementary construct focused on expectations rather than decisions, as in case of commitment.

Hope in the context of romantic relationships, particularly in therapy, remains even less operationalized and empirically differentiated. Moreover, there is a lack of brief, psychometrically sound instruments specifically designed to assess hope toward the partner and relationship, and limited evidence regarding its incremental predictive validity beyond established relationship constructs.

## 3. Methodology

### 3.1. The Present Study

The aim of the present study was to develop and validate a concise measure of hope for romantic relationships. Given that couple assessment often involves administering multiple instruments—making the process time-consuming—the present scale was designed to be brief, efficient, and easy to administer, while maintaining strong psychometric properties.

### 3.2. Participants

The sample consisted of 525 participants involved in a romantic relationship, with no missing data across the assessed variables. The mean age was 30.84 years (SD = 10.63), ranging from 18 to 64 years. The average relationship duration was 9.18 years (SD = 8.90), with a median of 5 years. The gender distribution was relatively balanced, with 54.9% identifying as female and 45.1% as male. Most participants had no children (57.5%), while the average number of children was 0.79 (SD = 1.25). Among those with children (n = 254), the mean age of the youngest child was 8.07 years (SD = 8.51). The majority of participants lived in their own household (79.0%), while 13.7% lived with their own parents and 7.2% with their partner’s parents. In terms of education, most participants had completed high school (45.1%) or university studies (36.4%). Participants’ financial status was generally perceived as moderate, with 70.9% reporting that they had exactly what they needed. Only a small proportion of participants (6.5%) reported having attended couples therapy at least once.

Although the sample size was adequate, EFA and CFA were conducted on the same dataset, which may inflate model fit and limit the generalizability of the findings. Future studies should test the factor structure using independent samples to ensure cross-validation. Given the simplicity of the model (four items, one factor), the risk of overfitting is reduced, but replication remains necessary.

### 3.3. Measures

*Hope for the romantic relationship*—The HRR scale (Marici, 2025a) was used, wherein recurring patterns related to individuals’ expectations about the future of their romantic relationships were identified (see Appendix A). The initial item pool was generated to capture core aspects of hope as expressed in therapeutic contexts. Given the applied nature of the construct, particular emphasis was placed on brevity and clarity, resulting in a short set of items suitable for both research and practical assessment. The items were subsequently refined to ensure conceptual coherence and ease of understanding, following the standard recommendations for scale development (DeVellis & Thorpe, 2021).

*Triangular love scale*—This measure was created by Sternberg R. J. in 1997 and it is one of the most known measures of romantic love (Sternberg, 1997). Although the scale consists of three dimensions, in the present study we were interested in the commitment subscale. This has 15 items and is measured on a 9-point Likert scale. Participants typically respond to a series of statements such as: “*I feel close to my partner*” or “*I am committed to maintaining this relationship*”. The alpha Cronbach for the scale was 0.919.

*Control of personal life*—This measures a partner’s control of the activities of personal life. It consists of six items measured on a scale from 0 (disagreement) to 5 (agreement) (Marici, 2025a). The items require participants to indicate how their partner treats them. The items take the following example form: “*Discourages me from meeting with my friends.”* The alpha Cronbach for the control of personal life scale was 0.900.

*Psychological aggression*—This consists of six items measured on a scale from 0 (disagreement) to 5 (agreement) (Marici, 2025a). “*When something happens that they don’t like, they pressure me to give in.*” The alpha Cronbach for the psychological aggression scale was 0.908.

*Pre-supplementing*—The term “supplementing” in a committed relationship refers to the behaviors oriented to people or activities that are intended to compensate partially or totally for unmet emotional needs and a perceived lack of love within the relationship, often in a manner of duplicity, secrecy and/or lying, neglecting the partner, and creating perceived difficulties in their relationship, which occur after the relationship begins to deteriorate. One form of supplementing is pre-supplementing, which refers to signaling one’s availability as a romantic partner while being in a committed relationship. The term was introduced within the framework of the Love and Rejection Messages Theory (LRM^T^) (Marici, 2025b). Pre-supplementing should be distinguished from proto-extradyadic behaviors. While both involve early orientations toward alternative partners, proto-extradyadic behaviors refer broadly to low-intensity actions that may precede extradyadic involvement. In contrast, pre-supplementing specifically involves signaling romantic availability as a response to unmet needs within the relationship. The scale asked partners to indicate whether “these activities appeared or intensified after you started to have misunderstandings with your romantic partner”. It consists of eleven items measured on a scale between 0 and 2: 0 (not the case), 1 (appeared), 2 (intensified) (Marici, 2025a, 2025b). Items are of the following type: “*I have given my phone number or address to someone I am attracted to.*” The alpha Cronbach for the scale was 0.969.

The dependent variables (psychological aggression, control of personal life, and pre-supplementing) were selected because they represent distinct maladaptive relational behaviors: coercive interaction (aggression), restriction of autonomy (control), and orientation toward alternative partners (pre-supplementing). These outcomes are theoretically linked to diminished expectations about the relationship’s future, making them appropriate criteria for testing whether hope explains variance beyond commitment.

### 3.4. Development of Hope for Romantic Relationship

The scale development followed several steps (DeVellis & Thorpe, 2021; Worthington & Whittaker, 2006). The process began with a conceptual definition of the construct and its differentiation from related constructs. The construct was defined as a future-oriented expectation comprising both cognitive and emotional components regarding the continuity and quality of the romantic relationship. Subsequently, an initial pool of items was generated based on theoretical frameworks and clinical observations. Initially there were six candidate items, but the item “*I am willing to build the relationship with my partner*.” was dropped as being too general.

A pilot study was conducted on-site on a small sample to evaluate the initial set of items (N = 31) on paper. The participants provided feedback on item clarity and comprehensibility, and minor revisions were made where needed. The wording of items was refined to ensure simplicity and redundant or ambiguous items were removed. The response format was also reviewed to confirm that it was intuitive and easy to use. Additionally, the overall flow and length of the questionnaire were assessed to reduce fatigue and improve engagement.

### 3.5. Questionnaire Administration

The data were collected using online questionnaires in Google Forms which were distributed to students as part of their assignment in multiple universities in Romania. They had to disseminate the questionaries to couples they know. Data collection was done between October 2025 and January 2026.

### 3.6. Statistical Analyses

Statistical analyses were conducted to examine the psychometric properties of the HRR scale. An exploratory factor analysis (EFA) was first performed to identify the underlying structure of the items, followed by a confirmatory factor analysis (CFA) to test the proposed one-factor model. Internal consistency was assessed using Cronbach’s alpha and McDonald’s omega coefficients. Convergent and discriminant validity were evaluated through correlations with relationship commitment. Finally, criterion-related validity was examined using correlation and regression analyses.

With 525 participants and four variables, the participant-to-variable ratio was approximately 131:1, substantially exceeding the commonly recommended minimum ratios (e.g., 5:1 or 10:1), thus supporting the adequacy of the sample size for analysis in R (version 4.5.3.). The software used was SPSS (version 26), R (version 4.5.3.) and Excel files.

The database is published online on ResearchGate at the following link: https://www.researchgate.net/publication/403085503_Hope_in_Romantic_Relationships_Development_and_Validation_of_a_Brief_Scale (accessed on 25 March 2026).

### 3.7. Ethics

The study was conducted in accordance with established ethical guidelines for research involving human participants. All participants were informed about the purpose of the study and provided their voluntary informed consent prior to participation. They were assured of the confidentiality and anonymity of their responses, and no identifying information was collected. Participation was entirely voluntary, and participants had the right to withdraw at any time without any consequences. The study complied with the ethical standards of the institutional research committee and with the principles outlined in the Declaration of Helsinki and was approved by the “1 Decembrie 1918” University of Alba Iulia, No. R-CE 47/24.03.2026.

### 3.8. Results

#### 3.8.1. Descriptive Statistics

A series of nonparametric analyses were conducted to examine the relationship between the HRR total score and demographic variables (see Table 1). Spearman correlations indicated no significant associations between HRR total score and age, relationship duration, number of children, or age of the youngest child (all *p* > 0.40). A Mann–Whitney U test revealed a small but statistically significant difference in HRR total score across sex, W = 31,191, *p* = 0.036, although median values were identical across groups. Kruskal–Wallis tests indicated no significant differences across living arrangement, education, or financial situation (all *p* > 0.25). However, a significant difference was found for previous psychological help, χ^2^(1) = 20.12, *p* < 0.001, suggesting that HRR total score varies as a function of prior engagement with psychological services.

The distribution of HRR scores was highly concentrated at the upper end of the scale, suggesting that participants generally report high levels of relational functioning (see Table 2). This pattern may reflect both the characteristics of the sampled population and the sensitivity of the instrument in capturing high-functioning relational dynamics rather than pathological variation.

A Chi-square test of independence indicated a significant association between maximum HRR scores and prior engagement with psychological services, χ^2^(1, N = 525) = 16.02, *p* < 0.001, φ = 0.17 (see Table 3).

Individuals without prior psychological experience were substantially more likely to report maximum HRR scores, suggesting a tendency toward idealized relationship evaluations. In contrast, participants with prior engagement in psychological services exhibited more variability, exhibiting greater critical awareness and nuanced evaluation of relational functioning. Although the effect size was small-to-moderate, it reflects a meaningful psychological difference in how relational functioning is perceived and reported. HRR is not merely a relationship score, but also reflects the level of idealization versus awareness.

#### 3.8.2. Correlations

Pearson correlations revealed that HRR was strongly positively associated with commitment (r = 0.76, *p* < 0.001) and strongly negatively associated with CPL (r = −0.51, *p* < 0.001) and PA (r = −0.57, *p* < 0.001). Additionally, HRR was negatively associated with pre-supplementing behaviors (r = −0.23, *p* < 0.001), indicating that higher relational functioning is linked to reduced engagement in alternative compensatory behaviors (see Table 4).

#### 3.8.3. Exploratory Factor Analysis

An exploratory factor analysis (EFA) based on polychoric correlations and using the minimum residual extraction method was conducted to examine the structure of the HRR scale. The Kaiser–Meyer–Olkin measure indicated good sampling adequacy (KMO = 0.80), and Bartlett’s test of sphericity was significant, χ^2^(6) = 2195.25, *p* < 0.001, supporting the factorability of the correlation matrix. A one-factor solution was extracted, explaining 81.3% of the variance. All items showed strong loadings on the latent factor, ranging from 0.84 to 0.96, indicating a highly coherent unidimensional structure (see Table 5).

#### 3.8.4. Reliability Analysis

The HRR scale demonstrated good internal consistency, with Cronbach’s α = 0.879 and McDonald’s ω = 0.888. Corrected item–total correlations ranged from 0.653 to 0.821, indicating that all items contributed adequately to the total scale score (see Table 6).

#### 3.8.5. Confirmatory Factor Analysis

**Figure 1:**
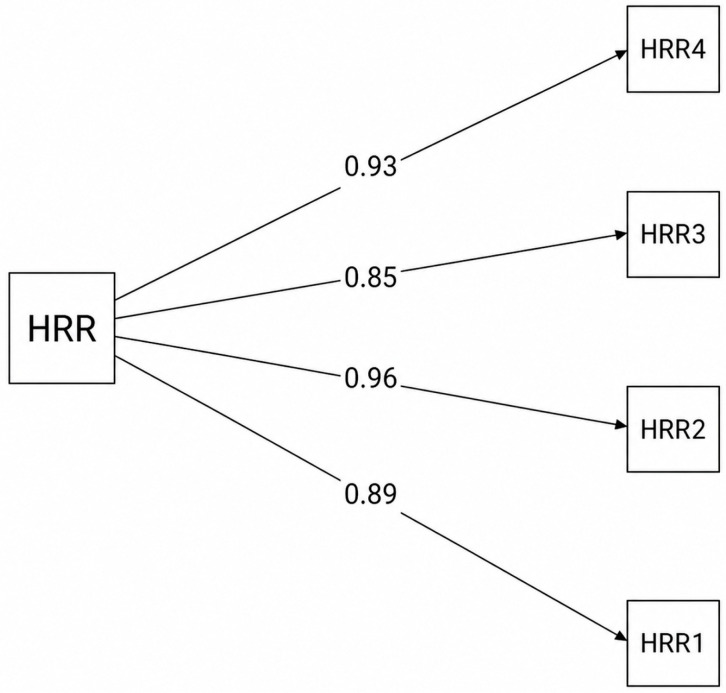
Standardized factor loadings for HRR for the entire sample.

A confirmatory factor analysis (CFA) using the WLSMV estimator was conducted to examine the unidimensional structure of the HRR scale. The model demonstrated a very good fit to the data, χ^2^(2) = 7.54, CFI = 0.999, TLI = 0.997, RMSEA = 0.073, SRMR = 0.029 (see Table 7). The standardized factor loadings were high, ranging from 0.85 to 0.96, indicating that all items strongly represented the latent construct. The uniqueness values ranged from 0.09 to 0.27, suggesting that most of the variance in the items was explained by the latent factor (see Table 8). These results support the reliability and unidimensionality of the HRR scale (see Figure 1).

#### 3.8.6. Descriptive Statistics and Convergent Validity on the Whole Group

The descriptive statistics indicated that participants reported relatively high levels of HRR (M = 18.84, SD = 2.69, N = 525) and commitment (M = 102.46, SD = 10.80, N = 461). Although HRR and commitment represent conceptually distinct constructs, they are theoretically related, as both reflect adaptive relational processes. Consistent with expectations, HRR was strongly and positively associated with commitment, *r* = 0.76, *p* < 0.001 (N = 461), supporting the construct validity of the scale. The magnitude of the correlation suggests that, although related, the constructs are not redundant, indicating that HRR represents a distinct but closely associated dimension of relationship functioning.

#### 3.8.7. Discriminant Validity

The HRR scale demonstrated excellent internal consistency (Cronbach’s α = 0.88, McDonald’s ω = 0.89, N = 461). Convergent validity was strongly supported by a high composite reliability (CR = 0.95) and average variance extracted (AVE = 0.82). Discriminant validity was also confirmed, as the square root of the AVE (0.91) exceeded the correlation with commitment (r = 0.76), and the AVE (0.82) was greater than the maximum shared variance (MSV = 0.58) (see Table 9). Overall, these findings provide strong support for the reliability and construct validity of the HRR scale.

#### 3.8.8. Criterion-Related Validity

Criterion-related validity was supported by a strong positive association between HRR and commitment, r(459) = 0.76, *p* < 0.001. Simple linear regression further showed that commitment significantly predicted HRR, F(1, 459) = 631.21, *p* < 0.001, explaining 57.9% of the variance in HRR (R^2^ = 0.579, adjusted R^2^ = 0.578) (see Table 10).

The unstandardized regression coefficient indicated that each one-unit increase in commitment was associated with a 0.154-point increase in HRR (B = 0.154, SE = 0.006, β = 0.76, t = 25.12, *p* < 0.001). The 95% confidence interval for the unstandardized coefficient ranged from 0.142 to 0.166.

These findings provide strong evidence for the criterion-related validity of the HRR scale. Because the model included a single predictor, multicollinearity diagnostics were not applicable.

#### 3.8.9. Measurement Invariance Across Gender—Multi-Group Confirmatory Factor Analysis (MG-CFA)

**Figure 2:**
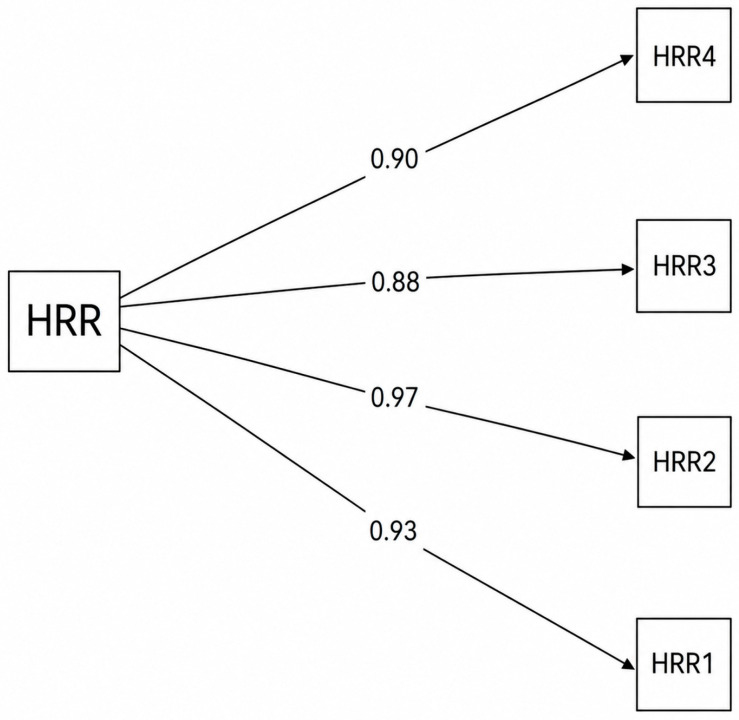
Standardized factor loadings for HRR for females.

A confirmatory factor analysis (CFA) using the WLSMV estimator was conducted on the female subsample (n = 288) to examine the structure of the HRR scale (see Figure 2). The one-factor model demonstrated an excellent fit to the data, χ^2^(2) = 3.62, CFI = 1.00, TLI = 0.999, RMSEA = 0.053, SRMR = 0.022. All items loaded strongly on the latent factor, with standardized loadings ranging from 0.88 to 0.97 (all *p* < 0.001), indicating a highly coherent and robust factor structure among female participants.

**Figure 3:**
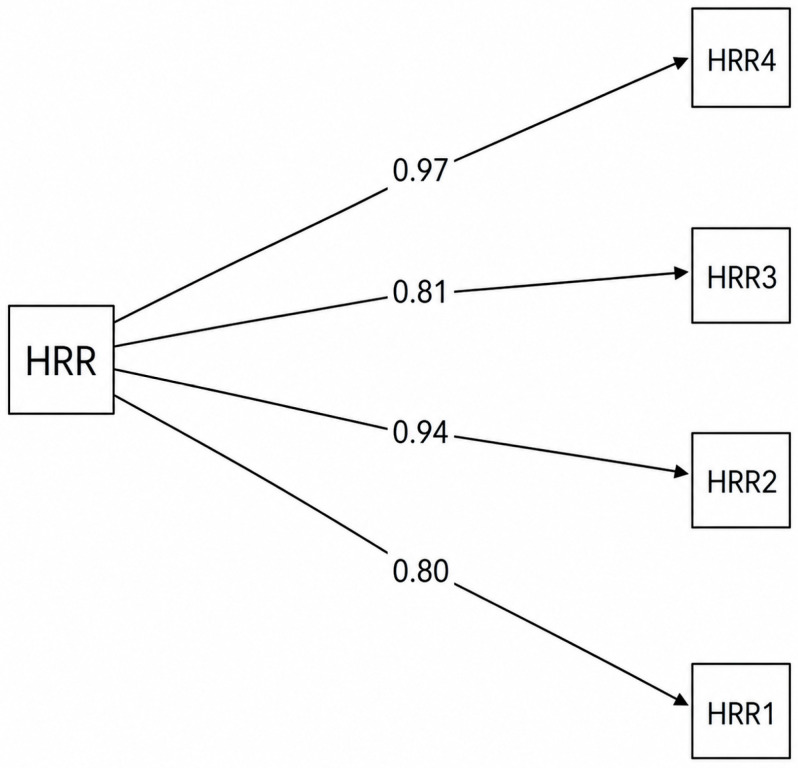
Standardized factor loadings for HRR for males.

A CFA was conducted on the male subsample (n = 237) using the same model specification (see Figure 3). The model demonstrated a very good fit to the data, χ^2^(2) = 5.44, CFI = 0.998, TLI = 0.995, RMSEA = 0.085, SRMR = 0.041. All factor loadings were high and statistically significant (all *p* < 0.001), ranging from 0.80 to 0.97, supporting the unidimensional structure of the scale in the male group.

#### 3.8.10. Predictive Validity and Incremental Predictive Validity

Predictive validity assesses whether a variable significantly predicts an outcome, whereas incremental predictive validity examines whether the variable explains additional variance beyond other relevant predictors (see Table 11).

For control of personal life (CPL), commitment significantly predicted the outcome in Step 1, accounting for 11.6% of the variance, F(1, 459) = 60.44, *p* < 0.001. When HRR was added in Step 2, the model significantly improved, ΔR^2^ = 0.067, ΔF(1, 458) = 37.50, *p* < 0.001, explaining a total of 18.3% of the variance. In the final model, HRR emerged as a significant predictor (β = −0.40, *p* < 0.001), whereas commitment was no longer significant. For psychological aggression (PA), commitment was a significant predictor in Step 1, explaining 16.0% of the variance, F(1, 459) = 87.19, *p* < 0.001. The addition of HRR in Step 2 significantly improved the model, ΔR^2^ = 0.077, ΔF(1, 458) = 46.30, *p* < 0.001, increasing the explained variance to 23.7%. In the final model, HRR remained a strong predictor (β = −0.43, *p* < 0.001), while commitment became non-significant.

For supplementing behaviors, commitment showed a small but significant effect in Step 1, explaining 2.4% of the variance, F(1, 459) = 11.52, *p* = 0.001. However, the addition of HRR in Step 2 did not significantly improve the model, ΔR^2^ = 0.005, ΔF(1, 458) = 2.54, *p* = 0.112. In the final model, neither commitment nor HRR significantly predicted supplementing behaviors.

## 4. Discussion

The present study provides robust evidence supporting the psychometric soundness of the hope for romantic relationship (HRR) scale as a concise and theoretically grounded measure of relational future orientation. The findings consistently indicate that HRR represents a coherent, unidimensional construct characterized by strong internal consistency and stable factor structure across analytic approaches and gender groups.

Importantly, the results extend beyond structural validation by demonstrating that HRR is meaningfully associated with, yet distinct from, relationship commitment. This distinction is theoretically significant (Snyder, 2002; Rusbult, 1980) as it differentiates between the motivational decision to maintain a relationship (commitment) and the cognitive–emotional expectation regarding its future (hope). In this sense, HRR captures a forward-looking evaluative dimension (Murray et al., 1996) that complements, rather than duplicates, existing constructs in romantic relationship science.

Furthermore, the findings provide clear support for the predictive and incremental validity of the HRR construct. Even when accounting for established relational dimensions, hope in the partner contributes uniquely to the explanation of key relationship outcomes.

From a theoretical perspective, these results align with contemporary models emphasizing the role of cognitive–affective representations in shaping relational trajectories (Dima-Cozma et al., 2014).

Another important aspect is related to the content of the final items. The elimination of the forgiveness item suggests that, although forgiveness is important for relationship functioning, it did not reflect the same latent dimension as the other items. In contrast, the retained items were more clearly focused on trust in the partner, the perceived suitability of the partner, the continuity of the relationship, and the expectation of a happy future together. This result suggests that hope for romantic relationships is centered more on future relational possibility and continuity, and less on broader adaptive behaviors such as forgiveness (Merolla, 2014; Snyder, 2002).

The fact that the distribution of HRR scores was highly concentrated at the upper end of the scale suggests that participants generally reported high levels of relational functioning. This pattern may reflect both the characteristics of the sampled population and the sensitivity of the instrument in capturing high-functioning relational dynamics rather than pathological variation. Overall, all of the analyses yielded satisfactory results, including a clear factor structure, strong item loadings, acceptable model fit indices, and consistent patterns of associations with related constructs. These findings indicate that the scale demonstrates good reliability and construct validity. Therefore, despite the presence of high scores, the instrument appears to adequately capture the intended construct and can be considered a psychometrically sound measure within the context of the present study.

The multi-group confirmatory factor analysis also supported the general adequacy of the one-factor model across genders. Although one item had a weaker loading in the male group, the overall structure remained stable, suggesting that the construct has a comparable meaning for both women and men. This is important because it supports the broader applicability of the scale, even if some items may function less consistently across groups and should be further examined in future studies on measurement invariance (Putnick & Bornstein, 2016).

From a practical point of view, the HRR scale may also have applied relevance. Because it is brief, coherent, and easy to administer, it may be useful not only in research, but also in counseling and couples therapy contexts. In these contexts, the way individuals perceive the future of the relationship may influence motivation for change, engagement in relational efforts, and openness to repair processes. Therefore, the direct assessment of hope may provide useful information beyond commitment (Merolla, 2014; Weingarten, 2010).

## 5. Conclusions

The aim of this study was to develop and validate a brief measure of hope for romantic relationships (HRR), conceptualized as a future-oriented expectation comprising both cognitive and emotional components regarding the continuity and quality of the romantic relationship. Additionally, the study sought to examine its reliability, construct validity, and predictive and incremental validity in relation to established relationship variables.

There are some limitations too. Firstly, the cross-sectional design of the study limits the ability to draw causal inferences regarding the relationships between hope concerning the romantic partner and other relational variables. Future research should employ longitudinal designs to examine the temporal stability and predictive directionality of the construct (Maxwell & Cole, 2007). Secondly, the reliance on self-report measures may introduce common method bias and social desirability effects, potentially inflating the observed associations between variables (Podsakoff et al., 2003). Incorporating multi-method approaches (e.g., partner reports or behavioral data) would strengthen the validity of the findings. Thirdly, although the scale demonstrated strong psychometric properties, the use of a relatively homogeneous sample may limit the generalizability of the results. Future studies should test the scale across more diverse populations and cultural contexts, beyond the Romanian context, to further establish its external validity. Future research should also test the scale on a clinical sample, where it is expected that the scale scores would be lower.

The findings also suggest that hope for romantic relationships represents a distinct future-oriented dimension of relationship functioning. Although it was strongly associated with commitment, the results indicate that hope is not fully reducible to the motivation to remain in the relationship. Rather, it seems to reflect the way individuals evaluate the continuity, stability, and future potential of the relationship with the romantic partner. In this sense, the present findings are consistent with theoretical perspectives emphasizing the role of future-oriented cognitions and relational expectations in close relationship maintenance (Baker et al., 2017; Lemay, 2016; Rusbult et al., 1998; Snyder, 2002).

From a practical point of view, the HRR scale may also be useful in both research and applied contexts. Because it is brief and easy to administer, it may offer a focused and quick way to assess how individuals perceive the future of their relationship and how open they are to relational investment. This may be relevant also for counseling and couples therapy, where perceptions regarding the future of the relationship may influence engagement in repair processes and motivation for change (Merolla, 2014; Weingarten, 2010). Future studies should continue to examine the predictive and incremental validity of the scale and test its functioning in more diverse relational contexts.

## Figures and Tables

**Table 1:** Associations between HRR total score and demographic variables.

Variable	Type	Test	Statistic	*p*	Effect
Age	Continuous	Spearman	ρ = 0.005	0.907	n.s.
Relationship duration	Continuous	Spearman	ρ = 0.000	0.996	n.s.
Youngest child age	Continuous	Spearman	ρ = 0.048	0.442	n.s.
Number of children	Continuous	Spearman	ρ = −0.009	0.829	n.s.
Sex	Categorical	Mann–Whitney	W = 31,191	0.036	*
Living arrangement	Categorical	Kruskal–Wallis	χ^2^(2) = 2.73	0.256	n.s.
Education	Categorical	Kruskal–Wallis	χ^2^(4) = 2.80	0.591	n.s.
Financial situation	Categorical	Kruskal–Wallis	χ^2^(4) = 4.10	0.393	n.s.
Previous psychological help	Categorical	Kruskal–Wallis	χ^2^(1) = 20.12	<0.001	***

Note. Spearman correlations were used for continuous variables. Mann–Whitney and Kruskal–Wallis tests were used for categorical variables. *p* < 0.05 *, *p* < 0.001 ***. n.s. = not significant.

**Table 2:** HRR total score by demographic factor.

Demographic Factors	Median (IQR)	*p*
**Overall HRR**	5 (4.75–5.00)	
**Sex**		
Female	5 (4.75–5.00)	
Male	5 (4.75–5.00)	0.036
**Living arrangements**		
With partner’s parents	5 (5.00–5.00)	
With own parents	5 (4.75–5.00)	
Own household	5 (4.75–5.00)	0.256
**Education**		
10 classes	5 (4.94–5.00)	
Doctorate	5 (5.00–5.00)	
University	5 (4.75–5.00)	
High school	5 (4.75–5.00)	
Master’s	5 (4.56–5.00)	0.591
**Financial status**		
Barely surviving	5 (4.88–5.00)	
Exactly what we need	5 (4.75–5.00)	
More than we need	5 (4.75–5.00)	
Less than we need	5 (4.25–5.00)	
Much more than we need	5 (5.00–5.00)	0.393
**Going to the therapist**		
Yes	4.75 (3.81–5.00)	
No	5 (4.75–5.00)	<0.001

Note: Median and interquartile range (IQR) were reported for the descriptive statistics. Differences between two groups were examined using Mann–Whitney U tests, while comparisons across multiple groups were conducted using Kruskal–Wallis tests.

**Table 3:** Association between maximum HRR scores and prior psychological service use.

HRR Maximum	Psychological Services (Yes)	Psychological Services (No)
Below maximum	61.8%	27.7%
Maximum (5)	38.2%	72.3%

**Table 4:** Pearson correlations among study variables.

Variable	1	2	3	4	5
1. Pre-supplementing	-	−0.16 **	0.18 ***	0.13 **	−0.23 ***
2. Commitment		-	−0.34 ***	−0.40 ***	0.76 ***
3. Control of personal life			-	0.72 ***	−0.51 ***
4. Psychological Aggression				-	−0.57 ***
5. HRR total score					-

Note. Values represent Pearson correlation coefficients (two-tailed). N = 525. *p* < 0.01 **, *p* < 0.001 ***.

**Table 5:** Factor loadings and communalities for the HRR scale.

Item	Loading	Communality
HRR1	0.86	0.74
HRR2	0.96	0.92
HRR3	0.84	0.71
HRR4	0.94	0.87

**Table 6:** Reliability indices and item–total correlations for the HRR scale.

Item	Item–Total Correlation
HRR1	0.653
HRR2	0.818
HRR3	0.714
HRR4	0.821

**Table 7:** Model fit indices for the HRR confirmatory factor analysis.

χ^2^	df	CFI	TLI	RMSEA	SRMR
7.54	2	0.999	0.997	0.073	0.029

**Table 8:** Standardized factor loadings and uniqueness for HRR items.

Item	Loading	Uniqueness
HRR1	0.885	0.216
HRR2	0.956	0.087
HRR3	0.853	0.272
HRR4	0.932	0.132

**Table 9:** Construct reliability and validity indices.

Construct	CR	AVE	√AVE	MSV
HRR	0.95	0.82	0.91	0.58

**Table 10:** Regression coefficients.

Predictor	Estimate (B)	SE	t	*p*	Standardized β	95% CI Lower	95% CI Upper
Constant	3.353	0.630	5.325	<0.001	-	2.116	4.590
Commitment	0.154	0.006	25.124	<0.001	0.761	0.142	0.166

**Table 11:** Incremental validity of the proposed scale over Sternberg’s dimensions (hierarchical regression).

Dependent Variable	Step	Predictors Entered	R^2^	ΔR^2^	F/ΔF	Significant Predictors (β, *p*)	VIF Range	Tolerance Range
Control of Per-sonal Life	Step 1	Commitment	0.116	-	F(1, 459) = 60.44 ***	Commitment (β = −0.34, *p* < 0.001)	-	-
	Step 2	Commitment, HRR	0.183	0.067	ΔF(1, 458) = 37.50 ***	HRR (β = −0.40, *p* < 0.001)	2.38–2.38	0.42
Psychological Aggression	Step 1	Commitment	0.160	-	F(1, 459) = 87.19 ***	Commitment (β = −0.40, *p* < 0.001)	-	-
	Step 2	Commitment, HRR	0.237	0.077	ΔF(1, 458) = 46.30 ***	HRR (β = −0.43, *p* < 0.001)	2.38–2.38	0.42
Supplementing	Step 1	Commitment	0.024	-	F(1, 459) = 11.52 **	Commitment (β = −0.16, *p* = 0.001)	-	-
	Step 2	Commitment, HRR	0.030	0.005	ΔF(1, 458) = 2.54 (ns)	-	2.38–2.38	0.42

Note. N = 461. Standardized coefficients (βs) are reported. ΔR^2^ represents the change in explained variance at each step. F and ΔF values correspond to the overall model and change in model fit, respectively. VIF = Variance inflation factor. *p* < 0.01 **, *p* < 0.001 ***.

## Data Availability

The database is available on request on ResearchGate: https://www.researchgate.net/publication/403085503_Hope_in_Romantic_Relationships_Development_and_Validation_of_a_Brief_Scale (accessed on 25 March 2026).

## Appendix A. Hope for Romantic Relationships (English and Romanian Versions) (Marici, 2025a)

Indicate how much you agree or disagree with these statements.

**Items Retained**	**Scale**
HRR 1. I feel that my partner is the most suitable person for me. *Simt că partenerul meu este cel mai potrivit pentru mine.*	Disagreement 0 1 2 3 4 5 Agreement Dezacord 0 1 2 3 4 5 Acord
HRR 2. I have great trust in my partner. *Am încredere în partenerul meu.*	Disagreement 0 1 2 3 4 5 Agreement Dezacord 0 1 2 3 4 5 Acord
HRR 3. I believe I will have a happy life with my partner moving forward. *Cred că voi avea o viață fericită cu partenerul meu de acum încolo.*	Disagreement 0 1 2 3 4 5 Agreement Dezacord 0 1 2 3 4 5 Acord
HRR 4. I believe I will stay with my partner for the rest of my life. *Cred că voi rămâne lângă el toată viața.*	Disagreement 0 1 2 3 4 5 Agreement Dezacord 0 1 2 3 4 5 Acord
**Dropped item**	
HRR 5. I am willing to forgive. *Sunt dispus să iert*.	Disagreement 0 1 2 3 4 5 Agreement Dezacord 0 1 2 3 4 5 Acord
Note: All rights are reserved to Marici Marius. This instrument can be used freely for research purposes only.	
